# Impact of established cardiovascular disease on 10-year death after coronary revascularization for complex coronary artery disease

**DOI:** 10.1007/s00392-021-01922-y

**Published:** 2021-08-25

**Authors:** Rutao Wang, Scot Garg, Chao Gao, Hideyuki Kawashima, Masafumi Ono, Hironori Hara, Robert-Jan van Geuns, Marie-Claude Morice, Piroze M. Davierwala, Arie Pieter Kappetein, David R. Holmes, William Wijns, Ling Tao, Yoshinobu Onuma, Patrick W. Serruys

**Affiliations:** 1 Department of Cardiology, Xijing Hospital, Xi’an, China; 2 Department of Cardiology, National University of Ireland, Galway (NUIG), P.O. University Road, Galway, H91 TK33 Ireland; 3 Department of Cardiology, Radboud University Medical Center, Nijmegen, The Netherlands; 4 East Lancashire Hospitals NHS Trust, Blackburn, Lancashire UK; 5 Department of Cardiology, Amsterdam Universities Medical Centers, Location Academic Medical Center, University of Amsterdam, Amsterdam, The Netherlands; 6 ICPS Ramsay-Generale de Sante, Massy, France; 7 Department of Cardiac Surgery, Heart Centre Leipzig, Leipzig, Germany; 8 Department of Cardiothoracic Surgery, Erasmus University Medical Centre, Rotterdam, The Netherlands; 9 Mayo Clinic, Rochester, MN USA; 10 NHLI, Imperial College London, London, UK

**Keywords:** CABG, Cardiovascular disease, Left main coronary artery disease, PCI, Three-vessel disease

## Abstract

**Aims:**

To investigate the impact of established cardiovascular disease (CVD) on 10-year all-cause death following coronary revascularization in patients with complex coronary artery disease (CAD).

**Methods:**

The SYNTAXES study assessed vital status out to 10 years of patients with complex CAD enrolled in the SYNTAX trial. The relative efficacy of PCI versus CABG in terms of 10-year all-cause death was assessed according to co-existing CVD.

**Results:**

Established CVD status was recorded in 1771 (98.3%) patients, of whom 827 (46.7%) had established CVD. Compared to those without CVD, patients with CVD had a significantly higher risk of 10-year all-cause death (31.4% vs. 21.7%; adjusted HR: 1.40; 95% CI 1.08–1.80, *p* = 0.010). In patients with CVD, PCI had a non-significant numerically higher risk of 10-year all-cause death compared with CABG (35.9% vs. 27.2%; adjusted HR: 1.14; 95% CI 0.83–1.58, *p* = 0.412). The relative treatment effects of PCI versus CABG on 10-year all-cause death in patients with complex CAD were similar irrespective of the presence of CVD (*p*_-interaction_ = 0.986). Only those patients with CVD in ≥ 2 territories had a higher risk of 10-year all-cause death (adjusted HR: 2.99, 95% CI 2.11–4.23, *p* < 0.001) compared to those without CVD.

**Conclusions:**

The presence of CVD involving more than one territory was associated with a significantly increased risk of 10-year all-cause death, which was non-significantly higher in complex CAD patients treated with PCI compared with CABG. Acceptable long-term outcomes were observed, suggesting that patients with established CVD should not be precluded from undergoing invasive angiography or revascularization.

**Trial registration:**

*SYNTAX*: ClinicalTrials.gov reference: NCT00114972. *SYNTAX Extended Survival***:** ClinicalTrials.gov reference: NCT03417050.

**Graphic abstract:**

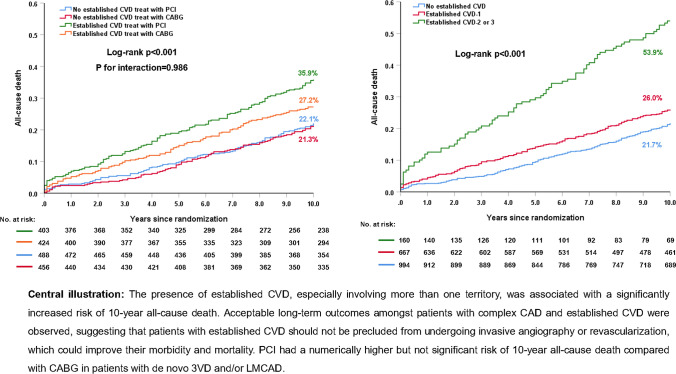

**Supplementary Information:**

The online version contains supplementary material available at 10.1007/s00392-021-01922-y.

## Introduction

Atherothrombosis is a systemic disease, usually involving more than one arterial bed, which has been termed poly-vascular disease [1, 2]. Patients with coronary artery disease (CAD) frequently have co-existing peripheral vascular disease (PVD) and/or cerebrovascular disease, with these patients at increased risk of in-hospital and mid-term adverse events [3–6]. However, to date, most available data are derived from cardiovascular prevention studies [7], and only limited data exist on outcomes following revascularization of patients with CAD and co-existing poly-vascular disease [8, 9]. Notably, these studies show that these vascular patients were less likely to undergoing invasive revascularization [10], and following percutaneous coronary intervention (PCI), they have a higher risk of short- and mid-term mortality [8, 9, 11, 12]. The impact of established cardiovascular disease (CVD) on very long-term all-cause death in patents with complex CAD following coronary revascularization also remains unclear. In addition, the optimal revascularization strategy for these patients has not been fully investigated.

The SYNTAX Extended Survival (SYNTAXES) study collected the 10-year survival status in 94% of the 1800 patients with de novo three-vessel disease (3VD) and/or left main coronary artery disease (LMCAD) who were originally randomised to PCI or coronary artery bypass grafting (CABG) in the SYNTAX trial [13]. Given the inclusion and exclusion criteria, the SYNTAX trial provides an enriched population to evaluate the effect of established CVD on outcomes after coronary revascularization in patients with complex CAD. The aims of the present study were, therefore, (1) to investigate the impact of established CVD on 10-year all-cause death following coronary revascularization in patients with complex CAD; (2) to examine the relative treatment effect of PCI versus CABG on 10-year all-cause death in patients with complex CAD and established CVD; and (3) to estimate the impact of the degree of established CVD on 10-year all-cause death.

## Methods

### Study design and population

The design and the primary results of the SYNTAX trial have been published previously [14–16]. Briefly, the SYNTAX trial (NCT00114972) was an international, multi-centre, randomised controlled trial which randomised all-comers patients with de novo 3VD and/or LMCAD, deemed eligible for both PCI and CABG, in a 1:1 fashion to either CABG (*n* = 897) or PCI (*n* = 903) with the TAXUS Express paclitaxel-drug eluting stents (Boston Scientific Corporation, Marlborough, MA, USA). The SYNTAX trial completed patient follow-up at 5 years [16]. The SYNTAXES study (NCT03417050) was an investigator-driven initiative that extended follow-up and aimed to evaluate vital status up to 10 years [13], funded by the German Heart Research Foundation (GHF; Frankfurt am Main, Germany). Follow-up was performed in accordance with local regulations of each participating centre and complied with the Declaration of Helsinki.

### Definitions and endpoints

In the present study, the cohort was stratified according to those with and without established CVD as reported by the investigator at the time of enrolment, and defined as ≥ 1 prior myocardial infarction (MI), prior cerebrovascular disease, or established PVD [1, 12]. The degree of established CVD was defined based on the extent of prior vascular disease with CVD-1 defined as patients having only one of a previous MI, cerebrovascular disease, or PVD; CVD-2 patients having two of these three conditions and CVD-3 patients having all three of these diagnoses. Only a few patients had vascular disease in three territories, so they were combined with patients in the CVD-2 group.

The primary endpoint of the SYNTAXES study was 10-year all-cause death. The secondary endpoint was all-cause death at maximum available follow-up. The 5-year rate of major adverse cardiovascular and cerebrovascular events (MACCE, defined as a composite endpoint of all-cause death, cerebrovascular accident, MI or repeat revascularization, the primary endpoint of the SYNTAX trial) according to the presence or absence of established CVD was also explored in the present study. Vital status was confirmed by contact with medical care personnel or by electronic healthcare record review and national death registries.

### Statistical analysis

Continuous variables are reported as mean ± standard deviation, and were compared using Student’s *t* test or Mann–Whitney *U* test. Categorical variables are shown as percentages and numbers, and were compared using Chi-square tests, or Fisher’s exact test when appropriate. The Kaplan–Meier method was used to estimate cumulative event rates, with the log-rank test used to assess differences between groups. Cox proportional hazards regression was used to calculate hazard ratios (HRs) with 95% confidence intervals (CI), and interaction tests were performed to assess the differences in the treatment effect of revascularization strategy in patients with and without established CVD. Multivariable analysis was performed to investigate whether established CVD was an independent predictor of all-cause death at 10 years. The Cox proportional hazards regression model included the following covariates: age, gender, body mass index, medically treated diabetes, hypertension, chronic obstructive pulmonary disease, left ventricular ejection fraction, creatinine clearance (ml/min), disease type (3VD or LMCAD), and the anatomical SYNTAX score, with all these variables selected based on prior knowledge of their association with clinical outcomes [17]. All analyses were performed using SPSS Statistics, version 25 (IBM Corp., Armonk, 281 N.Y., USA) and a *p* value of < 0.05 was considered statistically significant.

## Results

### Baseline and procedural characteristics

A total of 1800 patients were randomised in the SYNTAX trial, of which 29 had at least one missing piece of data on the status of co-existing established CVD. The study cohort, therefore, comprised of 1771 (98.3%) patients, of whom 827 (46.7%) had documented established CVD (Online Fig. 1). The median duration of follow-up was 11.2 years (IQR 7.9–12.1) overall and 11.8 years (11.0–12.3) in survivors. Patients with established CVD were older and had significantly higher rates of insulin treated diabetes, chronic obstructive pulmonary disease, renal impairment, congestive heart failure, and unstable angina. They had higher EuroSCOREs, Parsonnet scores, anatomical SYNTAX scores, and had more 3VD, lesions, total occlusions, bifurcations, stents, and had a longer total stent length (Online Table 1). They were less likely to have hypertension, had lower creatinine clearance and left ventricular ejection fraction (Online Table 1).

Patients with established CVD who underwent PCI were more likely to be female and had higher rates of hypertension and renal impairment compared to those underwent CABG (Table 1). Other baseline characteristics were well balanced between patients with established CVD treated with PCI or CABG.

**Table 1:** Baseline and procedural characteristics according to established CVD and revascularization strategy

	Established CVD (*n* = 827)			Without established CVD (*n* = 944)		
	PCI (*n* = 403)	CABG (*n* = 424)	*p* (PCI vs. CABG)	PCI (*n* = 488)	CABG (*n* = 456)	*p* (PCI vs. CABG)
Age (year)	66 ± 9.1	65.7 ± 9.7	0.588	64.5 ± 10.1	64.2 ± 9.7	0.562
Body mass index (kg/m^2^)	28.1 ± 5.1	27.6 ± 4.4	0.101	28.1 ± 4.5	28.3 ± 4.6	0.499
Gender			0.006			0.573
Female	25.8 (104/403)	17.9 (76/424)		21.9 (107/488)	23.5 (107/456)	
Male	74.2 (299/403)	82.1 (348/424)		78.1 (381/488)	76.5 (349/456)	
Medically treated diabetes	27.3 (110/403)	25.2 (107/424)	0.501	24 (117/488)	24.1 (110/456)	0.958
On insulin	13.2 (53/403)	12 (51/424)	0.626	7 (34/488)	8.6 (39/456)	0.362
Hypertension	66.5 (268/403)	59 (250/424)	0.025	70.9 (346/488)	68.6 (313/456)	0.449
Dyslipidemia	78.6 (315/401)	79.3 (334/421)	0.784	78.9 (381/483)	76.1 (343/451)	0.301
Current smoker	19.6 (79/403)	22.9 (96/420)	0.254	17.6 (86/488)	20.5 (93/453)	0.256
Chronic obstructive pulmonary disease	10.7 (43/403)	9.2 (39/424)	0.479	5.3 (26/488)	8.8 (40/456)	0.038
Impaired renal function	22.6 (91/403)	18.9 (80/424)	0.002	15.2 (74/488)	14 (64/456)	0.002
Creatinine clearance (ml/min)	84.6 ± 36.2	83.9 ± 30.7	0.776	88.4 ± 35.3	87.6 ± 28.1	0.714
Left ventricular ejection fraction	56.1 ± 13	55.7 ± 13.1	0.719	61.6 ± 12.1	60.8 ± 12.5	0.414
Congestive heart failure	5.5 (22/401)	8.2 (34/415)	0.126	2.7 (13/488)	2.4 (11/450)	0.832
Clinical presentation			0.599			0.850
Silent ischaemia	16.9 (68/403)	18.9 (80/424)		11.7 (57/488)	10.5 (48/456)	
Stable angina	47.1 (190/403)	48.1 (204/424)		65 (317/488)	65.6 (299/456)	
Unstable angina	36 (145/403)	33 (140/424)		23.4 (114/488)	23.9 (109/456)	
Euro SCORE	5 ± 2.7	4.8 ± 2.8	0.564	2.8 ± 2.1	2.8 ± 2.2	0.927
Parsonnet SCORE	9.3 ± 6.9	9.2 ± 7.2	0.789	7.9 ± 6.9	7.6 ± 6.3	0.559
Disease extent			0.136			0.451
3VD	61.5 (248/403)	66.5 (282/424)		59.2 (289/488)	56.8 (259/456)	
LMCAD	38.5 (155/403)	33.5 (142/424)		40.8 (199/488)	43.2 (197/456)	
Disease extent			0.342			0.318
LMCAD only	3.2 (13/403)	2.1 (9/424)		5.9 (29/488)	8.1 (37/455)	
LMCAD + 1VD	6 (24/403)	5.4 (23/424)		8.8 (43/488)	10.5 (48/455)	
LMCAD + 2VD	10.7 (43/403)	12 (51/424)		14.1 (69/488)	11.2 (51/455)	
LMCAD + 3VD	18.6 (75/403)	13.9 (59/424)		11.9 (58/488)	13.4 (61/455)	
2VD	1.7 (7/403)	1.2 (5/424)		2 (10/488)	3.1 (14/455)	
3VD	59.8 (241/403)	65.3 (277/424)		57.2 (279/488)	53.6 (244/455)	
Anatomical SYNTAX score	29.3 ± 11.6	29.3 ± 10.9	0.960	27.6 ± 11.3	28.8 ± 11.7	0.113
Number of lesions	4.6 ± 1.8	4.6 ± 1.7	0.965	4.1 ± 1.8	4.2 ± 1.9	0.436
Any total occlusion	0.3 ± 0.4	0.3 ± 0.4	0.866	0.2 ± 0.4	0.2 ± 0.4	0.252
Any bifurcation	0.7 ± 0.4	0.8 ± 0.4	0.423	0.7 ± 0.5	0.7 ± 0.5	0.859
Number of stents	4.8 ± 2.2	–		4.5 ± 2.3	–	
Total stent length per patient	90.3 ± 48.3	–		82.6 ± 47.2	–	
Off pump CABG	–	13 (55/424)		–	14.9 (68/456)	
Number of total conduits	–	2.8 ± 0.7		–	2.7 ± 0.7	
Number of arterial conduits	–	1.4 ± 0.6		–	1.4 ± 0.7	
Number of venous conduits	–	1.4 ± 0.9		–	1.3 ± 0.9	
LIMA use	–	80.7 (342/424)		–	83.3 (380/456)	
Complete revascularization	55.2 (222/402)	59.8 (244/408)	0.187	57.3 (276/482)	65.6 (292/445)	0.009

*CABG* coronary bypass artery grafting, *CVD* cardiovascular disease, *LMCAD* left main coronary artery disease, *PCI* percutaneous coronary intervention, *VD* vessel disease

### All-cause death according to established CVD

**Fig. 1:**
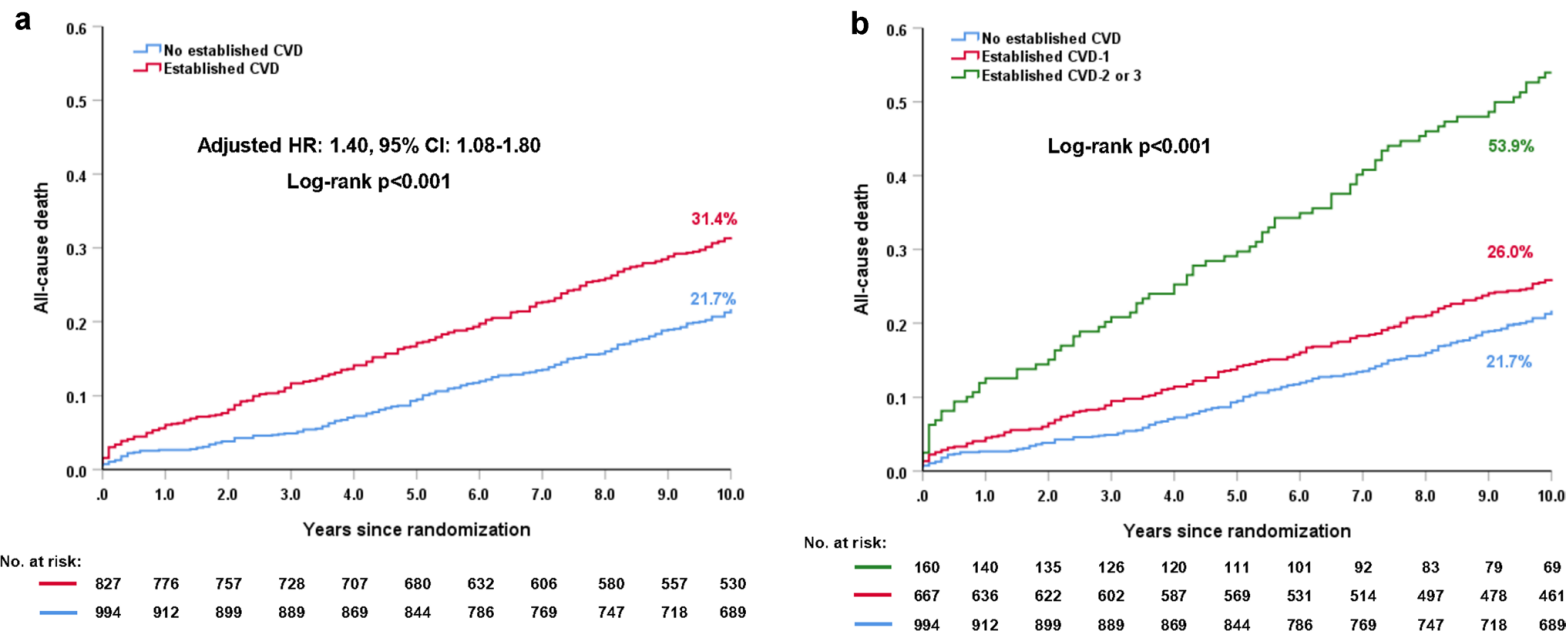
Ten-year all-cause death according to the extent of established CVD. **a** 10-year all-cause death in patients with established CVD versus those without; **b** 10-year all-cause death according to the extent of established CVD

All-cause death according to established CVD is shown in Table 2. Compared to those without a history of established CVD, patients with established CVD had a significantly higher risk of 10-year all-cause death (31.4% vs. 21.7%; adjusted HR: 1.40; 95% CI 1.08–1.80; *p* = 0.010, Fig. 1a). Similar results were observed at maximum follow-up (43.6% vs. 37.8%; adjusted HR: 1.42; 95% CI 1.23–1.80; *p* = 0.003). Landmark analysis showed that the presence of established CVD resulted in a higher risk of all-cause death at 5 years (16.9% vs. 9.3%; adjusted HR: 1.87; 95% CI 1.26–2.75; *p* = 0.002), and a numerically higher (but not significant) rate of all-cause death between 5 and 10 years (17.4% vs. 13.7%; adjusted HR: 1.10; 95% CI 0.78–1.55; *p* = 0.599).

**Table 2:** Clinical outcomes between patients with and without established CVD

	Without established CVD % (*n*/*N*)	Established CVD % (*n*/*N*)	Unadjusted HR 95% CI	*p*	Adjusted HR 95% CI	Adjusted *p*
0–5 years						
MACCE	30.4% (278/944)	35.3% (275/827)	1.18 (0.998–1.39)	0.053	1.20 (0.95–1.51)	0.122
Death, MI or stroke	15.3% (135/944)	24.0% (188/827)	1.69 (1.35–2.11)	< 0.001	1.56 (1.14–2.14)	0.005
All-cause death	9.3% (87/944)	16.9% (139/827)	2.05 (1.55–2.70)	< 0.001	1.87 (1.26–2.75)	0.002
Cardiac death	4.4% (39/944)	10.7% (80/827)	2.47 (1.68–3.62)	< 0.001	2.24 (1.30–3.84)	0.003
MI	5.4% (49/944)	8.8% (67/827)	1.64 (1.13–2.36)	0.008	1.69 (1.02–2.81)	0.044
Stroke	2.8% (25/944)	3.5% (26/827)	1.23 (0.71–2.14)	0.454	0.94 (0.42–2.09)	0.878
Revascularization	20.8% (183/944)	20.2% (146/827)	0.95 (0.76–1.18)	0.649	1.08 (0.81–1.45)	0.608
5–10 years						
All-cause death	13.7% (110/944)	17.4% (113/827)	1.31 (1.005–1.70)	0.045	1.10 (0.78–1.55)	0.599
10 years						
All-cause death	21.7% (197/944)	31.4% (252/827)	1.58 (1.31–1.90)	< 0.001	1.40 (1.08–1.80)	0.010
At maximum follow-up						
All-cause death	37.8% (246/944)	43.6% (310/827)	1.58 (1.33–1.86)	< 0.001	1.42 (1.23–1.80)	0.003

*CVD* cardiovascular disease, *MACCE* major adverse cardiovascular and cerebrovascular events, *MI* myocardial infarction

### All-cause death according to revascularization strategy

**Fig. 2:**
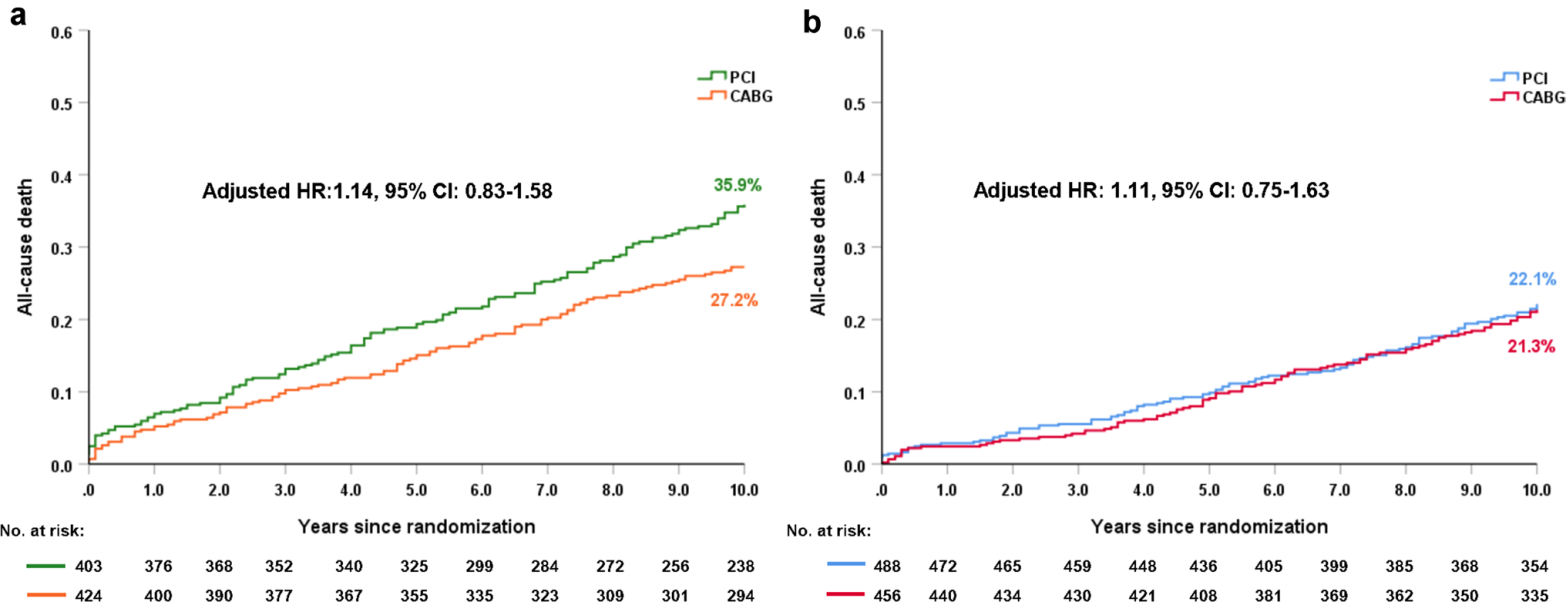
Ten-year all-cause death according to revascularization strategies and established CVD. **a** patients with established CVD; **b** patients without established CVD

In patients with established CVD, PCI had a higher crude rate of all-cause death at 10 years compared with CABG (35.9% vs. 27.2%; HR: 1.38; 95% CI 1.08–1.77, Log-rank *p* = 0.01). However, after adjustment for confounders, no significant difference between PCI and CABG was observed (adjusted HR: 1.14; 95% CI 0.83–1.58, *p* = 0.412, Table 3, Fig. 2a). In patients without established CVD, PCI had a comparable rate of 10-year all-cause death compared to CABG (22.1% vs. 21.3%, adjusted HR: 1.11; 95% CI 0.75–1.63, *p* = 0.601, Table 3, Fig. 2b). The risk of 10-year all-cause death was similar between PCI and CABG irrespective of the presence of established CVD (*p*_-interaction_ = 0.986, Table 3). Similar observations were found at maximum follow-up (Table 3).

**Table 3:** Clinical outcomes for established CVD versus no established CVD according to revascularization strategy

	Established CVD							Without established CVD							
Outcomes	PCI % (*n*/*N*)		CABG % (*n*/*N*)	HR: PCI/CABG (95% CI)	*p*	Adjusted HR 95% CI	Adjusted *p*	PCI % (*n*/*N*)	CABG % (*n*/*N*)	HR: PCI/CABG (95% CI)	*p*	Adjusted HR 95% CI	Adjusted *p*		Adjusted *p*_–interaction_
0–5 years															
MACCE	43.2% (168/403)		27.5% (107/424)	1.76 (1.38–2.24)	< 0.001	1.71 (1.24–2.36)	0.001	33.0% (159/488)	28.7% (119/456)	1.23 (0.97–1.56)	0.088	1.06 (0.76–1.46)	0.747		0.061
Death, MI or stroke	28.1% (111/403)		19.9% (77/424)	1.54 (1.15–2.05)	0.004	1.46 (0.99–2.17)	0.057	15.0% (72/488)	15.5% (63/456)	1.00 (0.71–1.41)	0.990	0.84 (0.52–1.35)	0.473		0.138
All-cause death	19.1% (77/403)		14.8% (62/424)	1.38 (0.98–1.94)	0.063	1.20 (0.78–1.87)	0.407	9.7% (47/488)	8.9% (40/456)	1.08 (0.70–1.68)	0.721	1.33 (0.71–2.49)	0.379		0.698
Cardiac death	13.3% (50/403)		8.3% (30/424)	1.73 (1.10–2.73)	0.016	1.53 (0.86–2.72)	0.149	5.7% (27/488)	2.9% (12/456)	2.01 (1.02–3.97)	0.040	5.06 (1.47–17.49)	0.010		0.064
MI	13.4% (50/403)		4.3% (17/424)	3.14 (1.81–5.45)	< 0.001	3.50 (1.66–7.41)	0.001	7.0% (33/488)	3.6% (16/456)	1.86 (1.03–3.39)	0.038	1.60 (0.71–3.61)	0.257		0.191
Stroke	3.6% (13/403)		3.3% (13/424)	1.03 (0.48–2.21)	0.948	0.54 (0.16–1.81)	0.313	1.5% (7/488)	4.2% (18/456)	0.34 (0.14–0.83)	0.012	0.18 (0.05–0.65)	0.009		0.377
Revascularization	28.9% (102/403)		11.7% (44/424)	2.57 (1.80–3.66)	< 0.001	2.24 (1.43–3.52)	< 0.001	25.0% (117/488)	16.0% (66/456)	1.68 (1.24–2.27)	0.001	1.40 (0.94–2.09)	0.095		0.117
5–10 years															
All-cause death	20.7% (63/403)		14.6% (50/424)	1.45 (1.00–2.10)	0.049	1.05 (0.65–1.69)	0.848	13.8% (57/488)	13.6% (53/456)	1.01 (0.69–1.47)	0.962	0.991 (0.61–1.61)	0.970		0.836
10 years															
All-cause death	35.9% (140/403)		27.2% (112/424)	1.38 (1.08–1.77)	0.010	1.14 (0.83–1.58)	0.412	22.1% (104/488)	21.3% (93/456)	1.05 (0.79–1.39)	0.742	1.11 (0.75–1.63)	0.601		0.986
At maximum follow-up															
All-cause death	46.0% (163/403)	41.2% (147/424)		1.26 (1.01–1.58)	0.041	1.09 (0.81–1.46)	0.580	36.7% (136/488)	39.6% (110/456)	1.16 (0.90–1.50)	0.238	1.07 (0.75–1.52)	0.711		0.997

*CVD* cardiovascular disease, *MACCE* major adverse cardiovascular and cerebrovascular events, *MI* myocardial infarction

### All-cause death according to anatomical SYNTAX score

In patients with established CVD, a numerically higher rate of all-cause death at 10 years and at maximum follow-up was seen following PCI compared with CABG in all anatomical SYNTAX score tertiles (Online Tables 2 and 3). In contrast, amongst patients without established CVD, there were no significant differences in all-cause death at 10 years and at maximum follow-up between PCI and CABG in any anatomical SYNTAX score tertile (Online Tables 2 and 3). No significant interaction was observed between the modality of revascularization and SYNTAX score tertile on 10-year all-cause death amongst patients with (*p*_-interaction_ = 0.472) or without established CVD (*p*_-interaction_ = 0.521, Online Table 2).

### All-cause death according to extent of established CVD

After adjustment for confounders, compared to those without established CVD, patients with one territory of CVD were found to have a trend for an increased risk of 10-year all-cause death, whilst the risk was a significant 2.9 times higher amongst those having more than one territory involved (Fig. 1b, Table 4). The HR for mortality decreased over time from 3.44 to 2.99 and then 2.97 at 5-year, 10-year and maximum follow-up, respectively (Table 4).

**Table 4:** All-cause death according to extent of established CVD

All-cause death	Unadjusted HR CVD-1/no CVD	*p* (CVD-1/no CVD)	Unadjusted HR CVD-2/no CVD	*p* (CVD-2/no CVD)	Adjusted HR CVD-1/no CVD	Adjusted *p* (CVD-1/no CVD)	Adjusted HR (CVD-2/no CVD)	Adjusted *p* (CVD-2/no CVD)
At 5 years	1.54 (1.15–2.07)	0.004	3.67 (2.57–5.23)	< 0.001	1.52 (1.00–2.31)	0.051	3.44 (2.09–5.68)	< 0.001
Between 5 and 10 years	1.03 (0.77–1.38)	0.835	2.89 (1.99–4.19)	< 0.001	0.88 (0.60–1.28)	0.508	2.65 (1.60–4.39)	< 0.001
At 10 years	1.26 (1.02–1.54)	0.029	3.23 (2.5–4.18)	< 0.001	1.12 (0.85–1.48)	0.421	2.99 (2.11–4.23)	< 0.001
At maximum follow-up	1.30 (1.08–1.56)	0.005	3.08 (2.43–3.91)	< 0.001	1.17 (0.91–1.51)	0.212	2.97 (2.14–4.15)	< 0.001

*CVD* cardiovascular disease, *HR* hazard ratio, *VD* vessel disease

After adjustment for confounders, prior MI was not an independent predictor of all-cause death, whereas both prior cerebrovascular disease and PVD were independent predictors of all-cause death at 10 years and at maximum follow-up (Table 5).

**Table 5:** All-cause death according to the three affected arterial beds

	Prior MI		Prior cerebrovascular disease		PVD	
	Adjusted HR (95% CI)	Adjusted *p*	Adjusted HR (95% CI)	Adjusted *p*	Adjusted HR (95% CI)	Adjusted *p*
At 5 years	1.29 (0.87–1.90)	0.203	1.50 (0.99–2.26)	0.053	2.91 (1.94–4.37)	< 0.001
At 10 years	1.06 (0.81–1.39)	0.670	1.63 (1.22–2.17)	0.001	2.45 (1.81–3.31)	< 0.001
At maximum follow-up	0.91 (0.71–1.18)	0.481	1.67 (1.28–2.19)	< 0.001	2.26 (1.69–3.01)	< 0.001

*MI* myocardial infarction, *PVD* peripheral vascular disease

### SYNTAX score II 2020 for predicting death at 10 years in patients with and without established CVD

**Fig. 3:**
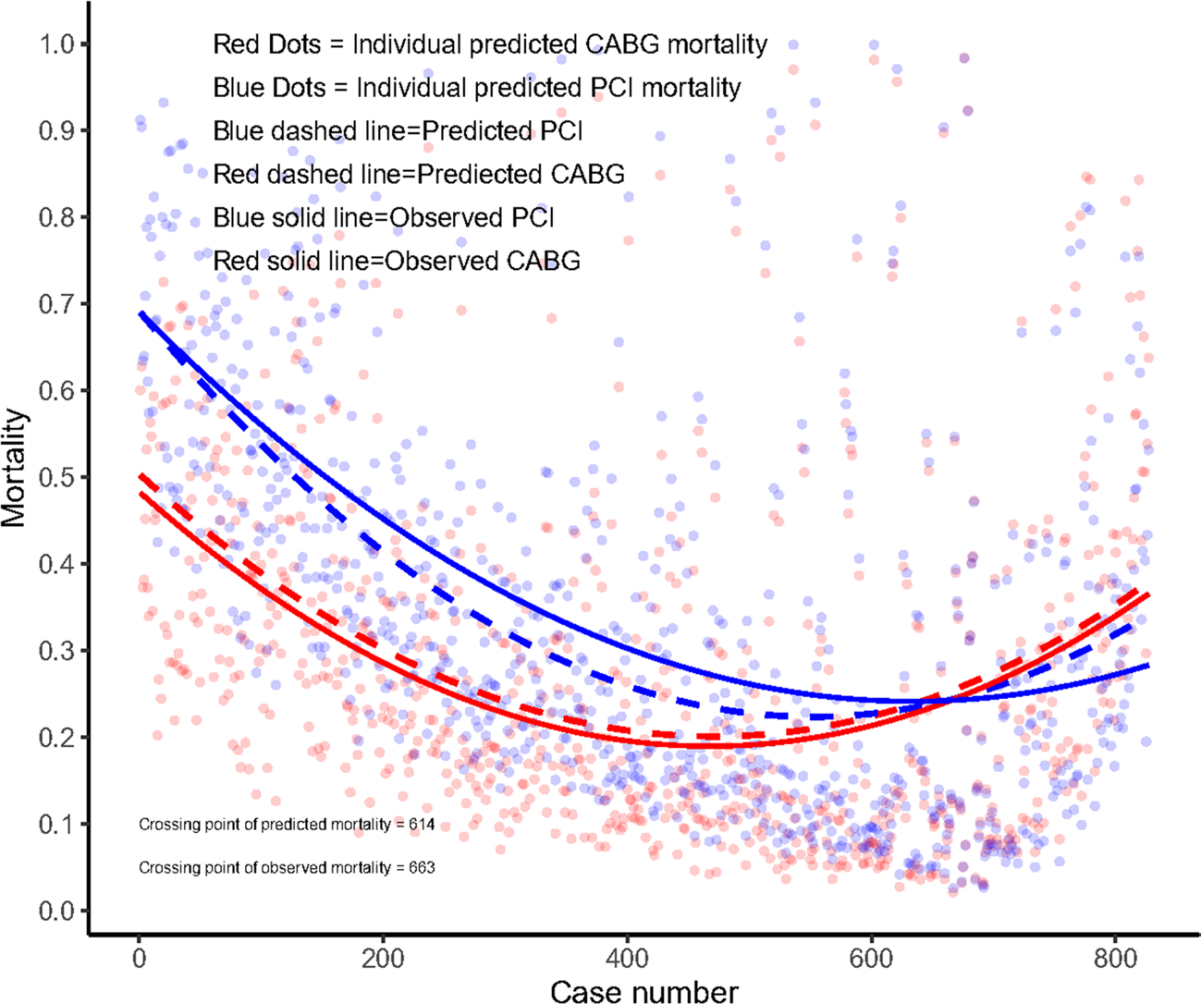
The individual difference between predicted mortality (dashed lines) using the SYNTAX Score II 2020 and the individual observed mortality (solid lines), between PCI and CABG in patients with established CVD. Blue dashed line represents the predicted mortality after PCI; red dashed line represents the predicted mortality after CABG; blue solid line represents the observed mortality after PCI; red solid line represents the observed mortality after CABG

Figure 3 shows ranked individual differences (*n* = 827) in predicted mortalities for patients with established CVD undergoing either PCI (blue dashed line) or CABG (red dashed line). In terms of ranking, there are 614 patients with higher predicted mortality after PCI than CABG, following which a crossover point in predicted mortalities (equipoise) is reached; beyond this point are 213 patients whose predicted mortality is lower with PCI than CABG. The solid lines in Fig. 3 depict, in a spline regression (LOESS) [18], the observed mortality after PCI or CABG. Notably, these observed mortalities crossover at the 663rd ranked patient suggesting only that specific patient had the same prognosis after PCI or CABG. The remaining 164 patients had higher observed mortality after surgery compared to PCI. Therefore, in contrast to the neutral “average treatment effect” observed in patients with established CVD at 10 years with either CABG or PCI, the SYNTAX score II 2020 clearly identifies individuals who derive a treatment-specific survival benefit.

## Discussion

In the present study, we assessed the impact of established CVD on 10-year all-cause death and evaluated the treatment effect of PCI versus CABG in patients with 3VD and/or LMCAD. The main findings are: (1) co-existing established CVD was common, being present in nearly half of the patients with 3VD and/or LMCAD; (2) patients with established CVD had a higher risk of all-cause death compared to those without, with the risk increasing according to the number of arterial beds affected; (3) the relative treatment effects of PCI versus CABG on 10-year all-cause death were similar irrespective of whether established CVD was present or not.

Given the common etiologies, it is not surprising that atherosclerosis frequently involves multiple vascular beds. In the REACH (The Reduction of Atherothrombosis for Continued Health) Registry, 15.9% of patients with symptomatic atherothrombosis had symptomatic poly-vascular disease [1]. The prevalence of co-existing established CVD in the current study is higher than that reported in prior randomised studies of PCI, PCI versus CABG and surgical registries [12, 19, 20]. This difference reflects the complexity of CAD which was required to be enrolled in the SYNTAX study, and suggests these patients may have a more malignant diffuse form of atherosclerosis with extensive widespread inflammation [21]. Consequently, the rates of statin and ACE-I/ARB use are disappointing, considering these are the cornerstones of managing atherosclerotic disease [22, 23]. Propensity-matched data comparing patients with LMCAD treated with CABG in the SYNTAX and EXCEL study have already hypothesised that the significant temporal improvements in the prescribing of secondary preventative therapies at discharge and 3-year follow-up in the EXCEL study drove many of the improved clinical outcomes observed in EXCEL [24].

Numerous studies have shown a correlation between the presence of established CVD and higher in-hospital and mid-term adverse outcomes following coronary revascularization [3, 4, 10, 12]. Consequently, although these patients represent a higher risk profile, studies indicate they are less likely to receive invasive management (coronary angiography or revascularization) [3, 10] with some reporting diagnostic cardiac catheterization rates of only 40–60% amongst older patients with non-STEMI and co-existing CVD [25]. Data on the impact of established CVD on very long-term all-cause death post-revascularization are limited, especially in patients with complex CAD, and to our knowledge, our study is the first to evaluate this.

Post-PCI, an observational study with a mean follow-up of 7.3 years showed that established CVD was associated with an increased risk of morbidity and mortality [11]. In the Global Leaders study, the largest PCI trial conducted to-date, patients with established CVD had higher rates of all-cause death, MI, stroke and revascularization with no significant differences in bleeding at 2 years [12]. Post-CABG, retrospective studies by Chu et al. and Nakamura et al. have both shown that symptomatic PVD is associated with poorer survival at 9- and 10-year follow-ups, respectively [26, 27]. Overall, after coronary revascularization (PCI or CABG), Morikami et al. found that established CVD was associated with higher adverse outcomes, which was mainly driven by the increased risk for non-coronary cardiovascular events [8]. In the BARI study, 5-year survival was 75.8% in patients with CVD and 90.2% for those without (*p* < 0.001) [20]. Despite these data, our study has shown acceptable adjusted long-term outcomes amongst patients with complex CAD and established CVD, which collectively represent a very high-risk population, suggesting that these patients should not be precluded from undergoing invasive angiography or coronary revascularization, which could improve their morbidity and mortality.

Our results show that the risk of mortality increases with the number of arterial beds involved, however, in our analysis, compared to those without established CVD, patients with only one affected arterial bed only had a trend for higher 10-year all-cause death, a result inconsistent with previous findings [3, 11, 28, 29]. Possible reasons for this discrepancy are multifactorial and include the differences in the enrolled populations, with our patients having very complex CAD, representing a higher risk population. The most plausible reason, however, is that prior MI (the commonest affected arterial bed amongst patients with established CVD in this study) was not found to be an independent predictor of all-cause death. Moreover, prior studies were mostly from large-scale observational studies, whereas our analysis, with a relatively limited sample size, may not have had adequate statistical power to detect the modest risk between groups. Studies with larger sample sizes in this high-risk population are, therefore, warranted to explore these outstanding issues. Finally, the HR for all-cause death in patients with more than one diseased arterial bed decreased over time (Table 4), indicating that established CVD increased the cumulative risk of all-cause death during the early part of follow-up but had less impact long term. These early events may be procedure related, with the reduced hazard over time reflective of the accumulated prognostic benefit of secondary preventative medications. The 10-year follow-up in the present study was much longer than prior studies, unveiling the decline in risk, which may have also potentially contributed to the comparable mortality between patients with only one affected arterial bed and those without.

Comparison between PCI and CABG in patients with established CVD has not been fully investigated. Observational data from patients with PVD and multi-vessel CAD showed that revascularization with CABG led to better adjusted 3-year survival than PCI [30]. In contrast, randomised data from the BARI study showed no difference in 5-year survival between patients with multi-vessel disease and established CVD treated with balloon angioplasty or CABG, however, this study was hampered by limited power to detect a treatment effect due to the small size of the established CVD subgroup (*n* = 303/1816) [20]. In the current analysis, we observed a higher risk of 5-year MACCE with PCI compared with CABG, which was mainly driven by the higher risk of MI and repeat revascularization (Table 3); no difference was observed in 5-year all-cause death. Similarly, at 10 years, PCI had a non-significant numerically higher risk of all-cause death compared with CABG. The SYNTAX score II 2020 was derived from the SYNTAXES population, and PVD was identified as a prognostic factor [31]. The non-significant difference in outcomes between PCI and CABG may also, therefore, be because PVD, which is only one component of established CVD, had a limited sample size (*n* = 175), such that the high crude mortality at 10 years did not remain significant after adjustment for confounders. More importantly, we found that in contrast to the neutral “average treatment effect” observed in patients with established CVD at 10 years with either CABG or PCI, the SYNTAX score II 2020 clearly identifies individuals who derive a treatment-specific survival benefit.

### Limitations

Although the SYNTAXES trial is one of the largest trials comparing PCI and CABG in complex CAD, it may not have adequate statistical power to produce reliable evidence for subgroup analyses [32]. There was no formal correction for multiple testing for subgroup analyses in the trial, considering the post hoc nature of the analysis [33]. Therefore, the reported results should be interpreted as exploratory and hypothesis-generating only. The randomisation in the SYNTAX trial was not stratified according to established CVD. Therefore, imbalances exist between groups. Although we performed adjustment for confounders, the inability to include all relevant confounders may cause bias that cannot be adjusted. Outcomes may have been affected by the location of the established CVD [10, 34]; however, our limited sample size precluded any meaningful comparisons between affected arterial beds. Studies with larger sample sizes in this high-risk population are warranted to explore these outstanding issues. The endpoint was all-cause death only. MACE and quality-adjusted life years (QUALY) are also relevant outcomes from the patient’s viewpoint [35]. However, all-cause death has been considered as the most robust and unbiased index for clinical assessment, and is less likely affected by ascertainment bias [36]. Finally, in the SYNTAX study, patients received PCI with the first-generation DES, the results are, therefore, only partially applicable to the contemporary new generation of DES. However, it is unavoidable that the findings from long-term follow-up data are based on outdated technology, whilst the evidence for contemporary technology can be derived only from short-term follow-up studies. Finally, an inherent bias may exist that patients with severe established CVD, which precludes performance of PCI, may not have been included in the randomised cohort and would have probably by default been included in the CABG registry in the SYNTAX trial.

## Conclusion

The presence of established CVD, especially involving more than one territory, was associated with a significantly increased risk of 10-year all-cause death. We observed acceptable long-term outcomes amongst patients with complex CAD and established CVD, suggesting that these patients should not be precluded from undergoing invasive angiography or revascularization, which could improve their morbidity and mortality. Overall, whilst there was a neutral treatment effect, the SYNTAX score II 2020 was able to identify those patients who would benefit the most from either CABG or PCI. The association between revascularization strategy and very long-term ischaemic and safety outcomes in this high-risk population needs further investigation in dedicated trials.

## Supplementary Information

Below is the link to the electronic supplementary material.

Supplementary file1 (DOCX 57 kb)

## Abbreviations

CABG: Coronary bypass artery grafting
CAD: Coronary artery disease
CVD: Cardiovascular disease
CI: Confidence interval
HR: Hazard ratio
LMCAD: Left main coronary artery disease
MACCE: Major adverse cardiovascular and cerebrovascular events
MI: Myocardial infarction
PCI: Percutaneous coronary intervention
PVD: Peripheral vascular disease
3VD: Three-vessel disease

## References

[1] Bhatt DLSP, Ohman EM, Hirsch AT, Ikeda Y, Mas JL, Goto S, Liau CS, Richard AJ, Röther J, Wilson PW, REACH Registry Investigators (2006). International prevalence, recognition, and treatment of cardiovascular risk factors in outpatients with atherothrombosis. JAMA.

[2] Fernandez-Friera L, Penalvo JL, Fernandez-Ortiz A (2015). Prevalence, vascular distribution, and multiterritorial extent of subclinical atherosclerosis in a middle-aged cohort: the PESA (Progression of Early Subclinical Atherosclerosis) Study. Circulation.

[3] Bhatt DL, Peterson ED, Harrington RA (2009). Prior polyvascular disease: risk factor for adverse ischaemic outcomes in acute coronary syndromes. Eur Heart J.

[4] Bhatt DLEK, Ohman EM, Hirsch AT, Goto S, Mahoney EM, Wilson PW, Alberts MJ, D'Agostino R, Liau CS, Mas JL, Röther J, Smith SC, Salette G, Contant CF, Massaro JM, Steg PG, REACH Registry Investigators (2010). Comparative determinants of 4-year cardiovascular event rates in stable outpatients at risk of or with atherothrombosis. JAMA.

[5] Jukema JW, Szarek M, Zijlstra LE (2019). Alirocumab in patients with polyvascular disease and recent acute coronary syndrome: ODYSSEY OUTCOMES Trial. J Am Coll Cardiol.

[6] Zeymer U, Parhofer KG, Pittrow D (2009). Risk factor profile, management and prognosis of patients with peripheral arterial disease with or without coronary artery disease: results of the prospective German REACH registry cohort. Clin Res Cardiol.

[7] Gutierrez JA, Aday AW, Patel MR, Jones WS (2019). Polyvascular disease: reappraisal of the current clinical landscape. Circ Cardiovasc Interv.

[8] Morikami Y, Natsuaki M, Morimoto T (2013). Impact of polyvascular disease on clinical outcomes in patients undergoing coronary revascularization: an observation from the CREDO-Kyoto Registry Cohort-2. Atherosclerosis.

[9] Miura T, Soga Y, Doijiri T (2013). Prevalence and clinical outcome of polyvascular atherosclerotic disease in patients undergoing coronary intervention. Circ J.

[10] Kobo O, Contractor T, Mohamed MO (2020). Impact of pre-existent vascular and poly-vascular disease on acute myocardial infarction management and outcomes: an analysis of 2 million patients from the National Inpatient Sample. Int J Cardiol.

[11] van der Meer MG, Cramer MJ, van der Graaf Y (2014). The impact of polyvascular disease on long-term outcome in percutaneous coronary intervention patients. Eur J Clin Invest.

[12] Garg S, Chichareon P, Kogame N (2020). Impact of established cardiovascular disease on outcomes in the randomized global leaders trial. Catheter Cardiovasc Interv.

[13] Thuijs DJFM, Kappetein AP, Serruys PW (2019). Percutaneous coronary intervention versus coronary artery bypass grafting in patients with three-vessel or left main coronary artery disease: 10-year follow-up of the multicentre randomised controlled SYNTAX trial. The Lancet.

[14] Ong AT, Serruys PW, Mohr FW (2006). The SYNergy between percutaneous coronary intervention with TAXus and cardiac surgery (SYNTAX) study: design, rationale, and run-in phase. Am Heart J.

[15] Serruys PWMM, Kappetein AP, Colombo A, Holmes DR, Mack MJ, Stahle E, Feldman TE, van den Brand M, Bass EJ, Van Dyck N, Leadley K, Dawkins KD, Mohr FW, Investigators S (2009). Percutaneous coronary intervention versus coronary-artery bypass grafting for severe coronary artery disease. N Engl J Med.

[16] Mohr FW, Morice M-C, Kappetein AP (2013). Coronary artery bypass graft surgery versus percutaneous coronary intervention in patients with three-vessel disease and left main coronary disease: 5-year follow-up of the randomised, clinical SYNTAX trial. The Lancet.

[17] Pocock SJ, McMurray JJV, Collier TJ (2015). Statistical controversies in reporting of clinical trials: part 2 of a 4-part series on statistics for clinical trials. J Am Coll Cardiol.

[18] Wang R, Serruys PW, Gao C (2021). Ten-year all-cause death after percutaneous or surgical revascularization in diabetic patients with complex coronary artery disease. Eur Heart J.

[19] Eagle KA, Rihal CS, Foster ED, Mickel MC, Gersh BJ (1994). Long-term survival in patients with coronary artery disease: importance of peripheral vascular disease. The Coronary Artery Surgery Study (CASS) Investigators. J Am Coll Cardiol.

[20] Sutton-Tyrrell K, Rihal C, Sellers MA (1998). Long-term prognostic value of clinically evident noncoronary vascular disease in patients undergoing coronary revascularization in the Bypass Angioplasty Revascularization Investigation (BARI). Am J Cardiol.

[21] Libby P, Ridker PM, Hansson GK, Leducq Transatlantic Network on A (2009). Inflammation in atherosclerosis: from pathophysiology to practice. J Am Coll Cardiol.

[22] Libby P, Buring JE, Badimon L (2019). Atherosclerosis. Nat Rev Dis Primers.

[23] Hibi K, Kimura T, Kimura K (2011). Clinically evident polyvascular disease and regression of coronary atherosclerosis after intensive statin therapy in patients with acute coronary syndrome: serial intravascular ultrasound from the Japanese assessment of pitavastatin and atorvastatin in acute coronary syndrome (JAPAN-ACS) trial. Atherosclerosis.

[24] Modolo R, Tateishi H, Miyazaki Y, Pighi M, Abdelghani M, Roos MA, Wolff Q, Wykrzykowska JJ, de Winter RJ, Piazza N, Richardt G, Mohamed Abdel-Wahab O, Soliman YO, Van Mieghem NM, Serruys PW (2019). Quantitative aortography for assessing aortic regurgitation after transcatheter aortic valve implantation: results of the multicentre ASSESS-REGURGE Registry. EuroIntervention.

[25] Subherwal S, Bhatt DL, Li S (2012). Polyvascular disease and long-term cardiovascular outcomes in older patients with non-ST-segment-elevation myocardial infarction. Circ Cardiovasc Qual Outcomes.

[26] Nakamura T, Toda K, Miyagawa S (2016). Symptomatic peripheral artery disease is associated with decreased long-term survival after coronary artery bypass: a contemporary retrospective analysis. Surg Today.

[27] Chu D, Bakaeen FG, Wang XL (2008). The impact of peripheral vascular disease on long-term survival after coronary artery bypass graft surgery. Ann Thorac Surg.

[28] Alberts MJ, Bhatt DL, Mas JL (2009). Three-year follow-up and event rates in the international REduction of Atherothrombosis for Continued Health Registry. Eur Heart J.

[29] Volis I, Saliba W, Jaffe R, Eitan A, Zafrir B (2020). Effect of cerebrovascular and/or peripheral artery disease with or without attainment of lipid goals on long-term outcomes in patients with coronary artery disease. Am J Cardiol.

[30] O'Rourke DJ, Quinton HB, Piper W (2004). Survival in patients with peripheral vascular disease after percutaneous coronary intervention and coronary artery bypass graft surgery. Ann Thorac Surg.

[31] Takahashi K, Serruys PW, Fuster V (2020). Redevelopment and validation of the SYNTAX score II to individualise decision making between percutaneous and surgical revascularisation in patients with complex coronary artery disease: secondary analysis of the multicentre randomised controlled SYNTAXES trial with external cohort validation. Lancet.

[32] Rothwell PM (2005). Treating individuals 2. Subgroup analysis in randomised controlled trials: importance, indications, and interpretation. Lancet.

[33] Li G, Taljaard M, Van den Heuvel ER (2017). An introduction to multiplicity issues in clinical trials: the what, why, when and how. Int J Epidemiol.

[34] Achterberg S, Cramer MJ, Kappelle LJ (2010). Patients with coronary, cerebrovascular or peripheral arterial obstructive disease differ in risk for new vascular events and mortality: the SMART study. Eur J Cardiovasc Prev Rehabil.

[35] Stolker JM, Spertus JA, Cohen DJ (2014). Rethinking composite end points in clinical trials: insights from patients and trialists. Circulation.

[36] Lauer MSBE, Young JB, Topol EJ (1999). Cause of death in clinical research: time for a reassessment?. J Am Coll Cardiol.

