# Supplementary material for: Impact of established cardiovascular disease on 10-year death after coronary revascularization for complex coronary artery disease
Source: Clin Res Cardiol. 2021 Aug 25;110(10):1680–91. doi: 10.1007/s00392-021-01922-y (PMC8484091; doi:10.1007/s00392-021-01922-y)
Supplement: Supplementary file 1 — Supplementary file1 (DOCX 57 kb) [file 392_2021_1922_MOESM1_ESM.docx]

**Online table 1. Baseline characteristics according to established CVD.**

|  | **Established CVD (n=827)** | **Without established CVD (n=944)** | **P** |
| --- | --- | --- | --- |
| **Age (year)** | 65.85± 9.41 | 64.36± 9.9 | 0.001 |
| **Body mass index (kg/m^2^)** | 27.85± 4.75 | 28.16± 4.58 | 0.161 |
| **Gender** |  |  | 0.648 |
| Female | 21.8 (180/827) | 22.7 (214/944) |  |
| Male | 78.2 (647/827) | 77.3 (730/944) |  |
| **Medically treated diabetes** | 26.2 (217/827) | 24 (227/944) | 0.288 |
| On Insulin | 12.6 (104/827) | 7.7 (73/944) | 0.001 |
| **Hypertension** | 62.6 (518/827) | 69.8 (659/944) | 0.001 |
| **Dyslipidemia** | 79 (649/822) | 77.5 (724/934) | 0.467 |
| **Current smoker** | 21.3 (175/823) | 19 (179/941) | 0.241 |
| **Chronic obstructive pulmonary disease** | 9.9 (82/827) | 7 (66/944) | 0.027 |
| **Impaired renal function** | 20.7 (171/827) | 14.6 (138/944) | 0.004 |
| **Creatinine clearance (ml/min)** | 84.29± 33.6 | 88± 32.11 | 0.024 |
| **Left ventricular ejection fraction** | 55.9± 13.07 | 61.21± 12.28 | <0.001 |
| **Congestive heart failure** | 6.9 (56/816) | 2.6 (24/938) | <0.001 |
| **Clinical presentation** |  |  | <0.001 |
| Silent ischemia | 17.9 (148/827) | 11.1 (105/944) |  |
| Stable angina | 47.6 (394/827) | 65.3 (616/944) |  |
| Unstable angina | 34.5 (285/) | 23.6 (223/) |  |
| **Euro SCORE** | 4.89± 2.76 | 2.78± 2.14 | <0.001 |
| **Parsonnet SCORE** | 9.22± 7.07 | 7.77± 6.61 | <0.001 |
| **Disease extent** |  |  | 0.009 |
| 3VD | 64.1 (530/827) | 58.1 (548/944) |  |
| LMCAD | 35.9 (297/827) | 41.9 (396/944) |  |
| **Disease extent** |  |  | <0.001 |
| LMCAD only | 2.7 (22/ 827) | 7 (66/ 943) |  |
| LMCAD +1VD | 5.7 (47/ 827) | 9.7 (91/ 943) |  |
| LMCAD +2VD | 11.4 (94/ 827) | 12.7 (120/ 943) |  |
| LMCAD +3VD | 16.2 (134/ 827) | 12.6 (119/ 943) |  |
| 2VD | 1.5 (12/ 827) | 2.5 (24/ 943) |  |
| 3VD | 62.6 (518/ 827) | 55.5 (523/ 943) |  |
| **Anatomical SYNTAX score** | 29.32± 11.2 | 28.16± 11.53 | 0.033 |
| **Number of lesions** | 4.56± 1.72 | 4.18± 1.84 | <0.001 |
| **Any total occlusion** | 0.26± 0.44 | 0.21± 0.41 | 0.026 |
| **Any bifurcation** | 0.75± 0.43 | 0.7± 0.46 | 0.018 |
| **Number of stents** | 4.77± 2.22 | 4.46± 2.28 | 0.039 |
| **Total stent length per patient** | 89.7± 48.29 | 81.93± 47.2 | 0.016 |
| **Off pump CABG** | 6.8 (56/827) | 7.3 (69/944) | 0.376 |
| **Number of total conduits** | 2.79± 0.68 | 2.74± 0.73 | 0.300 |
| Number of arterial conduits | 1.38± 0.65 | 1.41± 0.66 | 0.462 |
| Number of venous conduits | 1.41± 0.87 | 1.32± 0.94 | 0.151 |
| **LIMA use** | 42 (347/827) | 40.8 (385/944) | 0.347 |
| **Complete revascularization** | 57.5 (466/810) | 61.3 (568/927) | 0.113 |

CABG: coronary bypass artery grafting; CVD: cardiovascular disease; LMCAD: left main coronary artery disease; VD: vessel disease.

**Online table 2. Ten-year all-cause death according to revascularization mode and SYNTAX score tercile.**

|  | **Established CVD** | | | |  |  | **Without established CVD** | | | |  |
| --- | --- | --- | --- | --- | --- | --- | --- | --- | --- | --- | --- |
|  | PCI | CABG | Adjusted HR 95% CI | Adjusted p | Adjusted p- _interaction_ |  | PCI | CABG | Adjusted HR 95% CI | Adjusted p | Adjusted p- _interaction_ |
| SYNTAX score | % (n/N) | % (n/N) |  |  |  |  | %(n/N) | % (n/N) |  |  |  |
| **0-22** | 23.6%  (27/120) | 20.1%  (23/118) | 0.80  (0.38-1.68) | 0.563 | 0.472 |  | 22.6%  (38/175) | 19.0%  (28/154) | 0.81  (0.42-1.56) | 0.528 | 0.521 |
| **23-32** | 35.8%  (51/146) | 28.8%  (44/157) | 1.16  (0.67-2.02) | 0.593 |  |  | 18.1%  (28/160) | 19.2%  (25/136) | 1.33  (0.56-3.16) | 0.519 |  |
| **≥33** | 46.6%  (62/137) | 31.0%  (44/147) | 1.19  (0.70-2.00) | 0.523 |  |  | 25.0%  (36/149) | 23.2%  (36/161) | 1.30  (0.72-2.34) | 0.386 |  |

**Online table 3. All-cause death at maximum follow-up according to revascularization mode and SYNTAX score tercile.**

|  | **Established CVD** | | | |  |  | **Without established CVD** | | | |  |
| --- | --- | --- | --- | --- | --- | --- | --- | --- | --- | --- | --- |
|  | PCI | CABG | Adjusted HR 95% CI | Adjusted p | Adjusted p- _interaction_ |  | PCI | CABG | Adjusted HR 95% CI | Adjusted p | Adjusted p- _interaction_ |
| SYNTAX score | % (n/N) | % (n/N) |  |  |  |  | %(n/N) | % (n/N) |  |  |  |
| **0-22** | 41.4%  (36/120) | 40.0%  (35/118) | 0.76  (0.39-1.49) | 0.418 | 0.403 |  | 37.3%  (50/175) | 41.4%  (37/154) | 0.90  (0.50-1.61) | 0.725 | 0.702 |
| **23-32** | 47.4%  (62/146) | 45.0%  (58/157) | 1.17  (0.71-1.92) | 0.539 |  |  | 34.5%  (38/160) | 40.9%  (30/136) | 1.16  (0.54-2.51) | 0.708 |  |
| **≥33** | 49.8%  (65/137) | 40.2%  (53/147) | 1.13  (0.69-1.87) | 0.621 |  |  | 33.8%  (45/149) | 27.6%  (39/161) | 1.28  (0.72-2.45) | 0.399 |  |

**Online Figure 1. Study flow chart.**

**
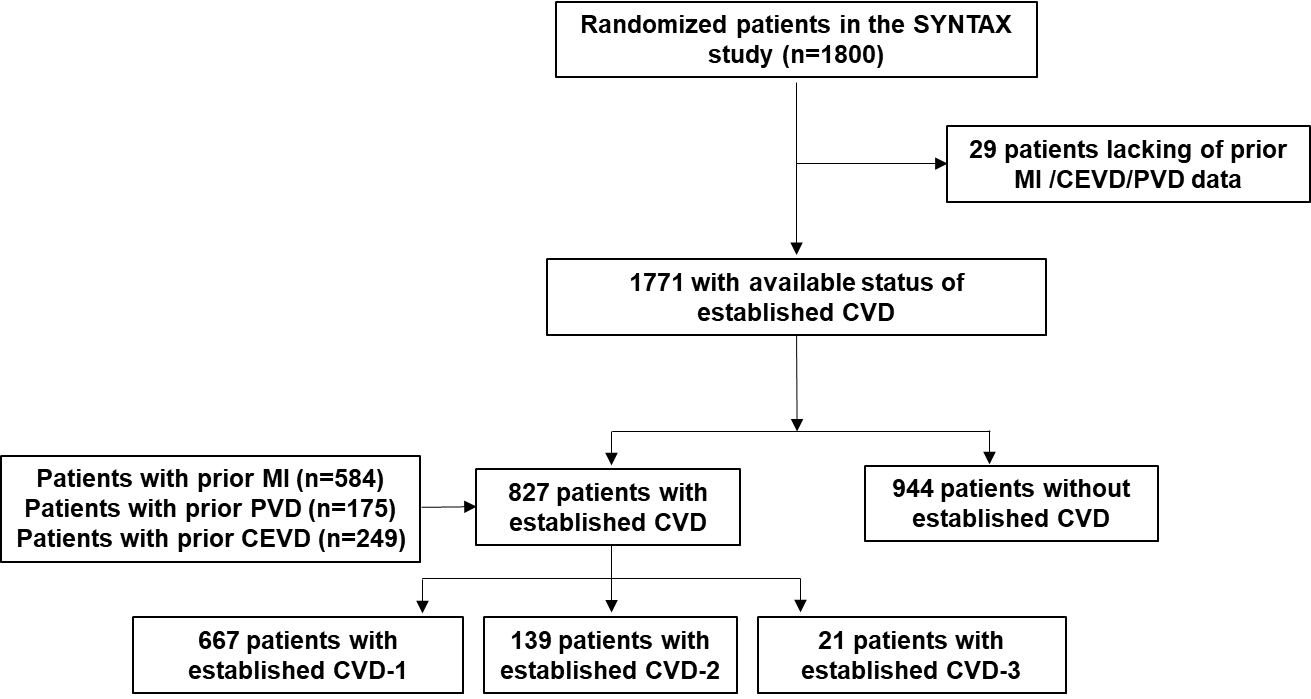
**

CEVD: cerebrovascular disease, CVD: cardiovascular disease, MI: myocardial infarction, PVD: peripheral vascular disease
